# Identification of Human Case of Avian Influenza A(H5N1) Infection, India

**DOI:** 10.3201/eid2806.212246

**Published:** 2022-06

**Authors:** Varsha Potdar, Megha Brijwal, Rakesh Lodha, Pragya Yadav, Santosh Jadhav, Manohar Lal Choudhary, Aashish Choudhary, Veena Vipat, Nivedita Gupta, Ashok Kumar Deorari, Lalit Dar, Priya Abraham

**Affiliations:** ICMR National Institute of Virology, Pune, India (V. Potdar, P. Yadav, S. Jadhav, M.L. Choudhary, V. Vipat, P. Abraham);; All India Institute of Medical Sciences, New Delhi, India (M. Brijwal, R. Lodha, A. Choudhary, A.K. Deorari, L. Dar);; Indian Council of Medical Research, New Delhi (N. Gupta)

**Keywords:** avian influenza, influenza A(H5N1), influenza, respiratory infections, viruses, zoonoses, India

## Abstract

A 11-year-old boy with acute myeloid leukemia was brought for treatment of severe acute respiratory infection in the National Capital Region, New Delhi, India. Avian influenza A(H5N1) infection was laboratory confirmed. Complete genome analysis indicated hemagglutinin gene clade 2.3.2.1a. We found the strain to be susceptible to amantadine and neuraminidase inhibitors.

Avian influenza viruses remain major threats worldwide, responsible for multiple outbreaks among poultry and episodes of transmission to humans. During January 2003–February 3, 2022, there were 862 reported cases of human infection with avian influenza A(H5N1) virus in 18 countries, resulting in a 53% case-fatality rate (https://www.who.int/docs/default-source/wpro---documents/emergency/surveillance/avian-influenza/ai-20220401.pdf).

The first outbreak of highly pathogenic avian influenza H5N1 in poultry in India, which occurred in January 2006 in Maharashtra, was caused by clade 2.2 (*1*); subsequent yearly outbreaks reported in poultry across the country were caused by newer clades 2.3.2.1 and 2.3.2.1c (*2**–**4*). Avian influenza surveillance in poultry revealed the presence of low-pathogenicity H9N2 and H4N6 viruses (*5*). On March 15, 2019, a human case of low-pathogenicity avian influenza A(H9N2) was detected in India (*6*). To date, 371 H5N1 and H5N8 avian influenza outbreaks in domestic or wild birds have been recorded in 15 of 28 states in India (https://empres-i.apps.fao.org/diseases). The first outbreaks of highly pathogenic avian influenza H5N8 in Europe were reported in August 2020 and since have been reported in poultry and wild birds in several countries in Europe, Asia, and Africa (https://web.oie.int/downld/SG/2021/A_88SG_2.pdf).

## The Study

An 11-year old boy who had acute myeloid leukemia diagnosed in June 2021 in the department of pediatrics at the All India Institute of Medical Sciences (AIIMS) in New Delhi was brought in for treatment of fever, cough, coryza, and breathing difficulty in the first week of July 2021. The patient was a resident of Gurugram, National Capital Region (Delhi), India. His clinical work-up showed febrile neutropenia with pneumonia and shock, which progressed to acute respiratory distress syndrome, so he was mechanically ventilated. He then developed multiorgan dysfunction, which ultimately resulted in his death on July 12, 2021. An in-depth interview with family members indicated that the patient often frequented a family-owned poultry business and may have been exposed to birds with undetected infection, although no infected domestic or wild avian sources or any environmental contamination had been reported in or around the residence of the child.

Staff in The AIIMS department of pediatrics referred nasopharyngeal (NP) swab specimens collected on July 7 and bronchoalveolar lavage (BAL) fluid collected on July 11 to the department of microbiology for respiratory virus testing, which used a Fast Track Diagnostics Respiratory Pathogens 21 kit and real-time PCR for influenza (https://www.siemens-healthineers.com) for diagnosis. Both BAL and NP samples tested positive for influenza A and influenza B Victoria lineage. Influenza A type could not be determined, so we referred samples to the National Influenza Centre at the Indian Council of Medical Research of the National Institute of Virology (Pune, India) for differential influenza diagnosis. The real-time reverse transcription PCR for avian influenza viruses H5Nx, H7N9, H9N2, and H10N8 was performed as documented elsewhere (*7**,**8*); results were positive for an H5Nx virus (cycle threshold value for H5 was 25). To confirm the subtype A/H5 identity, short fragments of multiple genes were sequenced: 230-bp matrix, 400-bp hemagglutinin (HA), 600-bp neuraminidase (NA), 600-bp nonstructural), and the influenza B HA gene, and analyzed results were analyzed using BLAST (https://blast.ncbi.nlm.nih.gov/Blast.cgi). We isolated and identified strain A/India/NIV-SARI-4571/2021 (H5N1) at a Biosafety Level 4 laboratory using MDCK cells.

We then generated whole-genome sequences from the original clinical NP samples and BAL-and MDCK-grown for passages 1 and 2 isolates using the Miniseq NGS Platform (Illumina, https://www.illumina.com) and a de-novo assembly program using QIAGEN CLC Genomics software 10.1.1 (https://www.quiagen.com). We constructed phylogenetic trees for 8 genes of A/India/NIV-SARI-4571/2021 (H5N1) virus using the neighbor-joining model with a Tamura-Nei nucleotide substitution performing 1,000 replicates of bootstrap support implemented in MEGA 7 (https://megasoftware.net) software. We submitted sequences to GenBank (accession nos. OL311384–91).

The sequences generated for the original clinical sample and the passaged virus were identical, suggesting no passage-induced mutations. We performed BLAST analysis of all 8 genes of A/India/NIV-SARI-4571/2021. The HA gene showed 97% identity with A/duck/Bangladesh/32003/2017 (H5N1). The polymerase basic (PB) 1 and nonstructural genes showed 97% nucleotide similarity to the avian influenza A/duck/Mongolia/729/2019 (H4N6) virus, suggesting probable reassortment.

**Figure 1:**
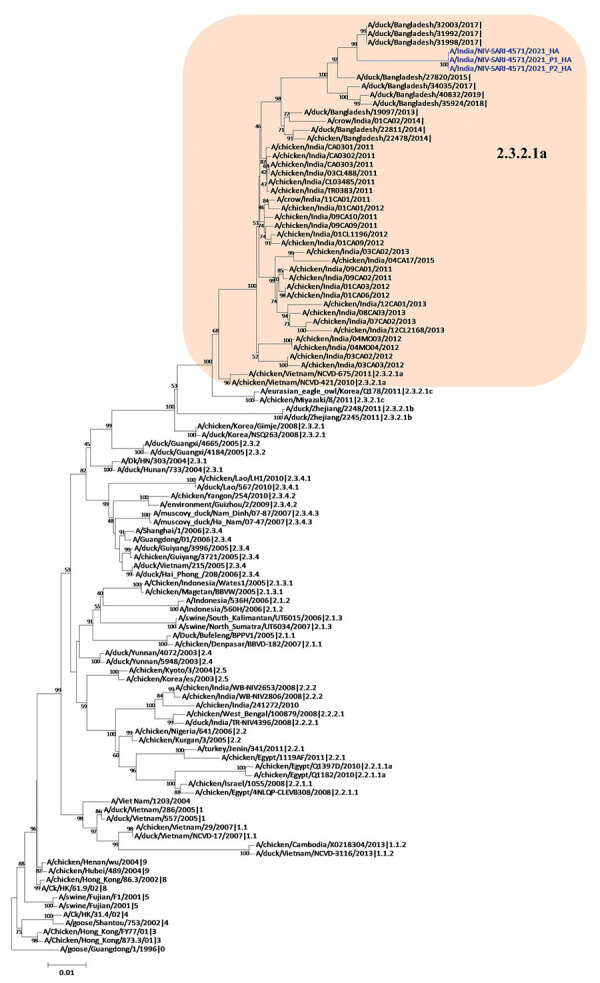
Hemagglutinin gene phylogenetic tree of avian influenza viruses, constructed using the neighbor-joining method as implemented in MEGA 7 (https://www.megasoftware.net). Blue text indicates the study strains (clinical and isolate); shaded area represents the Bangladesh and India strains in clade 2.3.2.1a. Gs/Guangdong/1/96 was used as the outgroup sequence. Scale bar indicates number of nucleotide substitutions per site.

**Figure 2:**
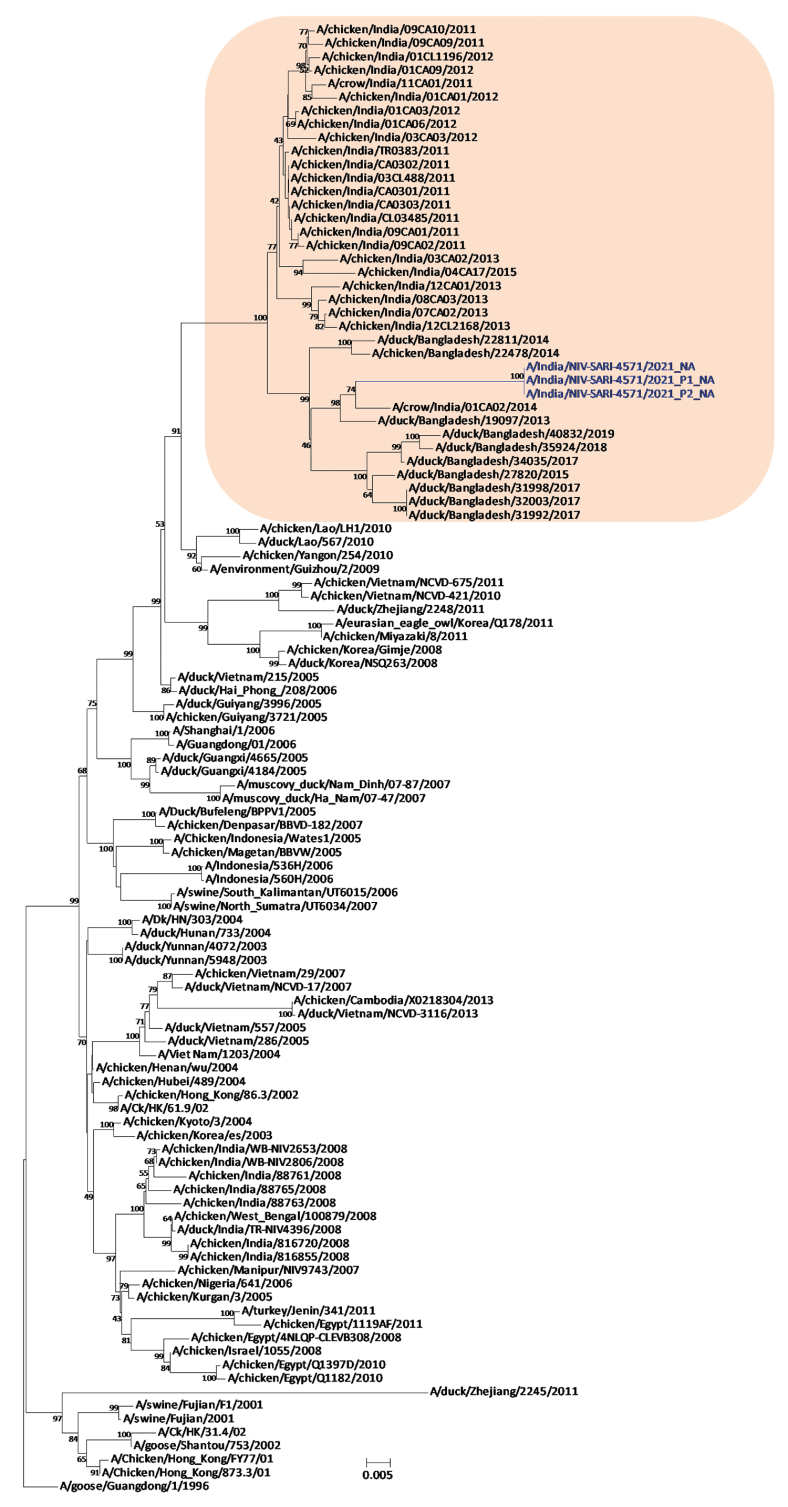
Neuraminidase gene phylogenetic tree constructed using the neighbor-joining method as implemented in MEGA 7 (https://www.megasoftware.net). Blue text indicates the study strains (clinical and isolate); shaded area represents the Bangladesh and India strains in clade 2.3.2.1a. Gs/Guangdong/1/96 was used as the outgroup sequence. Scale bar indicates number of nucleotide substitutions per site.

Phylogenetic analysis of the HA gene demonstrated that the virus belonged to clade 2.3.2.1a (Figure 1) and clustered with the A/duck/Bangladesh 2017 (H5N1)–like strain. Clade 2.3.2.1a has a H9N2-like PB1 gene and is the dominant clade in poultry in many countries, including Vietnam (*9*), India (*3*), Bangladesh, Bhutan, and Nepal (*10*). The NA gene clustered with an A/crow/India/01CA02/2014 (H5N1)–like strain (Figure 2). Phylogenetic trees indicate locations for the other 6 genes of the study strain (Appendix Figure 1). 

Using the WHO/CDC H5N1 inventory (*11*), we reviewed potential markers for the newly identified A/India/NIV-SARI-4571/2021 strain. The HA protein (H5) possessed a multiple basic amino acid cleavage site motif (PQRERRRKR*G), resulting in a highly pathogenic strain of clade 2.3.2.1 viruses. The sequence of the 220-loop receptor-binding site between amino acids Q222 and G224 remains conserved for the avian α 2–3 receptor.

We did not observe the NA and matrix 2 gene mutations responsible for neuraminidase inhibitors and amantadine resistance in the study strain. The virus remains purely avian-adapted, and we observed no markers for adaptation in mammals or pathogenicity for humans (Appendix Figure 2). We also did not observe PB2 hallmark mutations E627K and D701N, responsible for host adaptation and virulence in mammals in the study strain (*12*). The PDZ ligand domain (ESEV) at the C terminus remained conserved. We observed further compensatory mutations during the adaptation of H5N1 in mice, L89V, G309D, T339K, R477G, and I495V of PB2 (*13*) in the study strain. However, further studies are required to understand their role.

No evidence of H5 antibodies was detected in 18 close contacts of the deceased child. Available information and initial field investigations revealed that no additional cases were detected, indicating low human-to-human transmission. However, unreported high-pathogenicity avian influenza virus continues to exist in traded poultry in India, constituting a substantial risk for further human exposure (https://www.who.int/emergencies/disease-outbreak-news/item/human-infection-with-avian-influenza-a(h5n1)-%EF%BD%B0-india). Although widespread avian influenza outbreaks have been documented globally, only a limited number have shown transmission of avian influenza viruses to humans (*14*). The severe immunocompromised status of the child in this study may have made him vulnerable, and direct exposure to infected poultry might have been the source of infection. Influenza B virus, simultaneously detected with influenza A in this case-patient, is known to persist in such cases and might have been identified because of a prior infection (*15*).

## Conclusions

In December 2020 and early 2021, outbreaks of avian influenza H5N1 and H5N8 were reported in poultry in 15 states in India; the National Capital Region, Maharashtra, Punjab, and Kerala, in particular, were severely affected. The whole-genome sequencing of A/India/NIV-SARI-4571/2021 (H5N1) virus in our study provides valuable insight into the absence of hallmark mutations responsible for adaptation and virulence in mammals. The strain remained sensitive to amantadine and neuraminidase inhibitors. However, identification of a human H5N1 case in India highlights the need for systemic surveillance at the human–animal interface level.

Appendix Additional information about human case of avian influenza A(H5N1) infection in India.
