# Supplementary material for: Identification of Human Case of Avian Influenza A(H5N1) Infection, India
Source: Emerg Infect Dis. 2022 Jun;28(6):1269–73. doi: 10.3201/eid2806.212246 (PMC9155886; doi:10.3201/eid2806.212246)
Supplement: Appendix — Additional information about human case of avian influenza A(H5N1) infection in India. [file 21-2246-Techapp-s1.pdf]

Article DOI: https://doi.org/10.3201/eid2806.212246

Identification of Human Case of Avian Influenza A(H5N1) Infection, India

Appendix

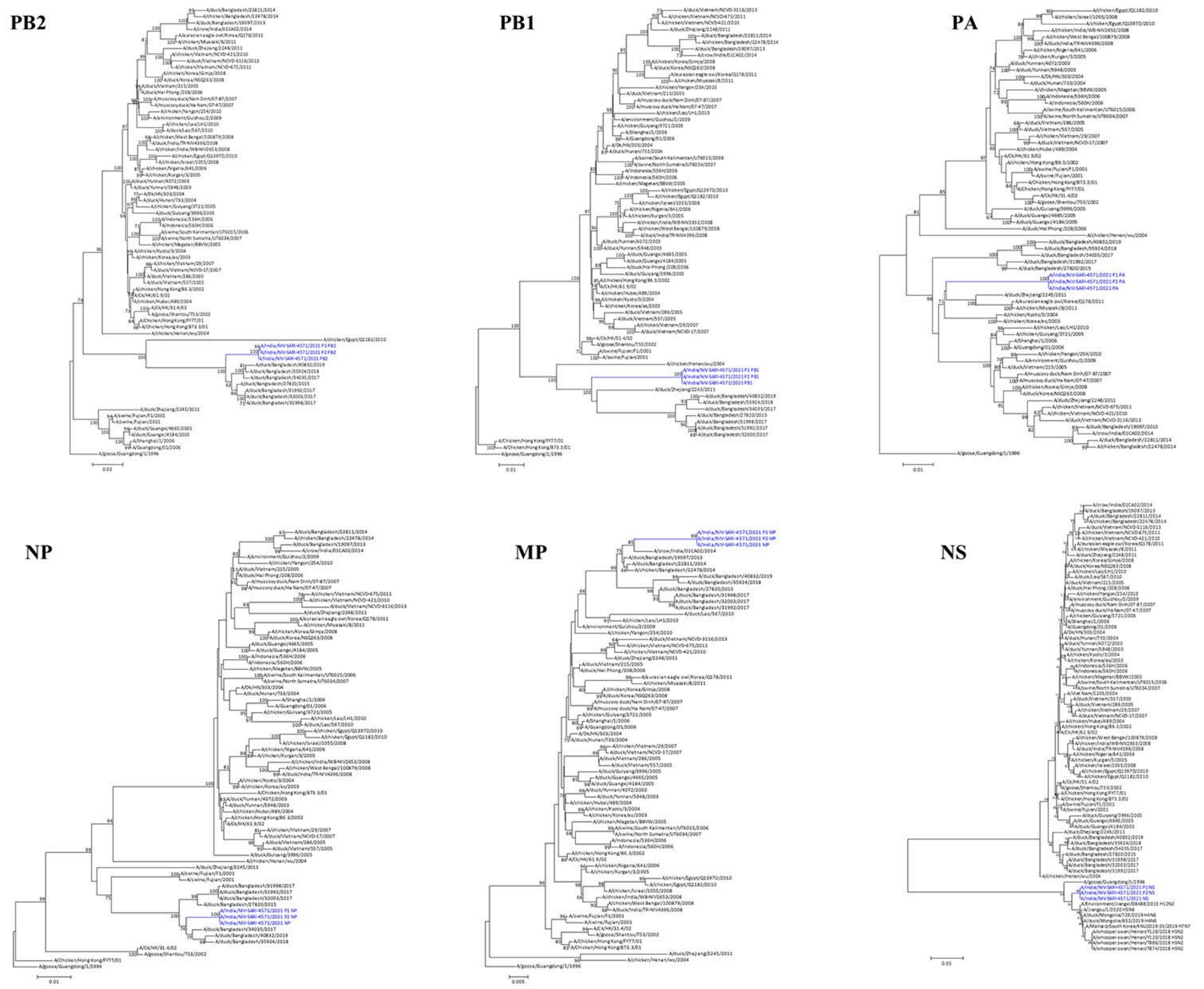

Appendix Figure 1. Phylogenetic tree of all 8 genes of study virus A/India/NIV-SARI-4571/2021 (H5N1): A) polymerase basic 2 gene (PB2); B) polymerase basic 1 gene (PB1); C) polymerase gene (PA); D) nuclear protein gene (NP); E) matrix gene (M); and F) nonstructural gene (NS). The numbers above the branches are the bootstrap probabilities (%) for each branch, determined using MEGA software, version 7 (https://megasoftware.net). We used Gs/Guangdong/1/96 as the out group.

```
#A/goose/Guangdong/1/1996            MERIKELRDL MSQSRTREIL TKTTVDHMAI IKKYTSGRQE KNPALRMKWM MAMKYPITAD KRIMEMIPER NEQGQTLWSK TNDAGSDRVM VSPLAVTWWN  [100]
#A/India/NIV-SARI-4571/2021_PB2      .......... .......... .......... .......... .......... .......... .......... .......... .......... ..........  [100]
#A/India/NIV-SARI-4571/2021_P1_PB2   .......... .......... .......... .......... .......... .......... .......... .......... .......... ..........  [100]
#A/India/NIV-SARI-4571/2021_P2_PB2   .......... .......... .......... .......... .......... .......... .......... .......... .......... ..........  [100]

#A/goose/Guangdong/1/1996            RNGPTTSTVH YPKVYKTYFE KVERLKHGTF GPVHFRNQVK IRRRVDINPG HADLSAKEAQ DVIMEVVFPN EVGARILTSE SQLTITKEKK EELQDCKIAP  [200]
#A/India/NIV-SARI-4571/2021_PB2      ......G.A. .......... .A........ .......... ......T... .......... .......... .......... .......... ..........  [200]
#A/India/NIV-SARI-4571/2021_P1_PB2   ......G.A. .......... .A........ .......... ......T... .......... .......... .......... .......... ..........  [200]
#A/India/NIV-SARI-4571/2021_P2_PB2   ......G.A. .......... .A........ .......... ......T... .......... .......... .......... .......... ..........  [200]

#A/goose/Guangdong/1/1996            LMVAYMLERE LVRKTRFLPV AGGTSSVYIE VLHLTQGTCW EQMYTPGGEV RNDDVDQSLI IAARNIVRRA TVSADPLASL LEMCHSTQIG GIRMVDILRQ  [300]
#A/India/NIV-SARI-4571/2021_PB2      .......... .......... .......... .......... ....------ ----...... .......... .......... .......... ..........  [300]
#A/India/NIV-SARI-4571/2021_P1_PB2   .......... .......... .......... .......... ........K. .......... .......... .......... .......... ..........  [300]
#A/India/NIV-SARI-4571/2021_P2_PB2   .......... .......... .......... .......... ........K. .......... .......... .......... .......... ..........  [300]

#A/goose/Guangdong/1/1996            NPTEEQAVDI CKAAMGLRIS SSFSFGGFTF KRTNGSSVKK EEEVLTGNLQ TLKIKVHEGY EEFTMVGRRA TAILRKATRR LIQLIVSGRD EQSIAEAIIV  [400]
#A/India/NIV-SARI-4571/2021_PB2      .......... .......... ........I. ...S.....R .......... ....R..... .......... .......... .......... ..........  [400]
#A/India/NIV-SARI-4571/2021_P1_PB2   .......... .......... ........I. ...S.....R .......... ....R..... .......... .......... .......... ..........  [400]
#A/India/NIV-SARI-4571/2021_P2_PB2   .......... .......... ........I. ...S.....R .......... ....R..... .......... .......... .......... ..........  [400]

#A/goose/Guangdong/1/1996            AMVFSQEDCM IKAVRGDLNF VNRANQRLNP MHQLLRHFQK DAKVLFQNWG IEPIDNVMGM IGILPDMTPS AEMSLRGVRV SKMGVDEYSS TERVVVSIDR  [500]
#A/India/NIV-SARI-4571/2021_PB2      .......... V......... .......... .......... .......... .....S.... ..V......N T......... .......... ..........  [500]
#A/India/NIV-SARI-4571/2021_P1_PB2   .......... V......... .......... .......... .......... .....S.... ..V......N T......... .......... ..........  [500]
#A/India/NIV-SARI-4571/2021_P2_PB2   .......... V......... .......... .......... .......... .....S.... ..V......N T......... .......... ..........  [500]

#A/goose/Guangdong/1/1996            FLRVRDQQGN VLLSPEEVSE TQGTEKLTIT YSSSMMWEIN GPESVLVNTY QWIIRNWETV KIQWSQDPTM LYNKMEFESF QSLVPKAARS QYSGFVRTLF  [600]
#A/India/NIV-SARI-4571/2021_PB2      .......R.. .......... .....R.... .......... .......... .......... .......... ........P. .........G ..........  [600]
#A/India/NIV-SARI-4571/2021_P1_PB2   .......R.. .......... .....R.... .......... .......... .......... .......... ........P. .........G ..........  [600]
#A/India/NIV-SARI-4571/2021_P2_PB2   .......R.. .......... .....R.... .......... .......... .......... .......... ........P. .........G ..........  [600]

#A/goose/Guangdong/1/1996            QQMRDVLGTF DTVQIIKLLP FAAAPPEPSR MQFSSLTVNV RGSGMRILVR GNSPVFNYNK ATKRLTVLGK DAGALTEDPD EGTAGVESAV LRGFLILGRE  [700]
#A/India/NIV-SARI-4571/2021_PB2      .......... .....V.... .......Q.. V......... .......... .......... .......... .....A.... .......... ........K.  [700]
#A/India/NIV-SARI-4571/2021_P1_PB2   .......... .....V.... .......Q.. V......... .......... .......... .......... .....A.... .......... ........K.  [700]
#A/India/NIV-SARI-4571/2021_P2_PB2   .......... .....V.... .......Q.. V......... .......... .......... .......... .....A.... .......... ........K.  [700]

#A/goose/Guangdong/1/1996            DKRYGPALSI NELSNLAKGE KANVLIMQGD VVLVMKRKRD FSILTDSQTA TKRIRMAIN*  [760]
#A/India/NIV-SARI-4571/2021_PB2      .......... .......... ......G... .......... S......... ..........  [760]
#A/India/NIV-SARI-4571/2021_P1_PB2   .......... .......... ......G... .......... S......... ..........  [760]
#A/India/NIV-SARI-4571/2021_P2_PB2   .......... .......... ......G... .......... S......... ..........  [760]
```

```
#A/goose/Guangdong/1/1996            MDVNPTLLFL KVPAQNAIST TFPYTGDPPY SHGTGTGYTM DTVNRTHQYS EKGKWTTNTE TGAPQLNPID GPLPEDNEPS GYAQTDCVLE AMAFLEESHP  [100]
#A/India/NIV-SARI-4571/2021_PB1      .......... .......... .......... .......... .......... .......... .......... .......... .......... ..........  [100]
#A/India/NIV-SARI-4571/2021_P1_PB1   .......... .......... .......... .......... .......... .......... .......... .......... .......... ..........  [100]
#A/India/NIV-SARI-4571/2021_P2_PB1   .......... .......... .......... .......... .......... .......... .......... .......... .......... ..........  [100]

#A/goose/Guangdong/1/1996            GIFENSCLET MEVVQQTRVD KLTQGRQTYD WTLKRNQPAA TALANTIEVF RSNGLTANES GRLIDFLKDV MESMDKGEME IITHFQRKRR VRDNMTKKMV  [200]
#A/India/NIV-SARI-4571/2021_PB1      .......... .......... .......... ...N...... .......... .......... .......R.. .D....E... .T........ ..........  [200]
#A/India/NIV-SARI-4571/2021_P1_PB1   .......... .......... .......... ...N...... .......... .......... .......R.. .D....E... .T........ ..........  [200]
#A/India/NIV-SARI-4571/2021_P2_PB1   .......... .......... .......... ...N...... .......... .......... .......R.. .D....E... .T........ ..........  [200]

#A/goose/Guangdong/1/1996            TQRTIGKKKQ RLNKRSYLIR ALTLNTMTKD AERGKLKRRA IATPGMQIRG FVYFVETLAR SICEKLEQSG LPVGGNEKKA KLANVVRKMM TNSQDTELSF  [300]
#A/India/NIV-SARI-4571/2021_PB1      .......... ...R...... .......... .......... .......... .......... .......... .......... .......... ..........  [300]
#A/India/NIV-SARI-4571/2021_P1_PB1   .......... ...R...... .......... .......... .......... .......... .......... .......... .......... ..........  [300]
#A/India/NIV-SARI-4571/2021_P2_PB1   .......... ...R...... .......... .......... .......... .......... .......... .......... .......... ..........  [300]

#A/goose/Guangdong/1/1996            TITGDNTKWN ENQNPRMFLA MITYITRNQP EWFRNVLSIA PIMFSNKMAR LGKGYMFESK SMKLRTQIPA EMLASIDLKY FNESTRKKIE KIRPLLIDGT  [400]
#A/India/NIV-SARI-4571/2021_PB1      .......... .......... .......... .......... .......... .......... ...I...... ....N.N... ..D..K.... ..........  [400]
#A/India/NIV-SARI-4571/2021_P1_PB1   .......... .......... .......... .......... .......... .......... ...I...... ....N.N... ..D..K.... ..........  [400]
#A/India/NIV-SARI-4571/2021_P2_PB1   .......... .......... .......... .......... .......... .......... ...I...... ....N.N... ..D..K.... ..........  [400]

#A/goose/Guangdong/1/1996            ASLSPGMMMG MFNMLSTVLG VSILNLGQKR YTKTTYWWDG LQSSDDFALI VNAPNHEGIE AGVDRFYRTC KLVGINMTKK KSYINRTGTC EFTSFFYRYG  [500]
#A/India/NIV-SARI-4571/2021_PB1      .......... .......... .......... .......... .......... .........Q .......... .......S.. .........F ..........  [500]
#A/India/NIV-SARI-4571/2021_P1_PB1   .......... .......... .......... .......... .......... .........Q .......... .......S.. .........F ..........  [500]
#A/India/NIV-SARI-4571/2021_P2_PB1   .......... .......... .......... .......... .......... .........Q .......... .......S.. .........F ..........  [500]

#A/goose/Guangdong/1/1996            FVANFSMELP SFGVSGINES ADMSIGVTVI KNNMMDNDLG PATAQMALQL FIKDYRYPYR CHRGDTQIQT RRSFELKKLW EQTRSKAGLL VSDGGPNPYN  [600]
#A/India/NIV-SARI-4571/2021_PB1      .......... .......... .......... ....IN.... .......... .......T.. .......... .......... .......... .......L..  [600]
#A/India/NIV-SARI-4571/2021_P1_PB1   .......... .......... .......... ....IN.... .......... .......T.. .......... .......... .......... .......L..  [600]
#A/India/NIV-SARI-4571/2021_P2_PB1   .......... .......... .......... ....IN.... .......... .......T.. .......... .......... .......... .......L..  [600]

#A/goose/Guangdong/1/1996            IRNLHIPEAG LKWELMDEDY QGRLCNPLNP FVSHKEIESV NNAVVMPAHG PAKSMEYDAV ATTHSWIPKR NRSILNTSQR GILEDEQMYQ KCCNLFEKFF  [700]
#A/India/NIV-SARI-4571/2021_PB1      ........VC .......... .......... .......... .......... .......... .......... .......... .......... ...S......  [700]
#A/India/NIV-SARI-4571/2021_P1_PB1   ........VC .......... .......... .......... .......... .......... .......... .......... .......... ...S......  [700]
#A/India/NIV-SARI-4571/2021_P2_PB1   ........VC .......... .......... .......... .......... .......... .......... .......... .......... ...S......  [700]

#A/goose/Guangdong/1/1996            PSSSYRRPVG ISSMVEAMVS RARIDARIDF ESGRIKKEEF AEIMKICSTI EELGRQK* [758]
#A/India/NIV-SARI-4571/2021_PB1      .......... .......... .......... .......... .......... ...R.... [758]
#A/India/NIV-SARI-4571/2021_P1_PB1   .......... .......... .......... .......... .......... ...R.... [758]
#A/India/NIV-SARI-4571/2021_P2_PB1   .......... .......... .......... .......... .......... ...R.... [758]

#A/goose/Guangdong/1/1996            MEQEQDTPWT QSTEHINIQK RGNGQRTQRL EHPNSIRLMD HYLRIMSRVG MHKQIVYWKQ WLSLKNPTQG SLKTRVLKRW KLFSKQEWIS * [91]
#A/India/NIV-SARI-4571/2021_PB1      .......... .......T.. .E...Q.... .....TQ... ....TTNQAD .....AC... ..F..S.... ....L..... RS......TN . [91]
#A/India/NIV-SARI-4571/2021_P1_PB1   .......... .......T.. .E...Q.... .....TQ... ....TTNQAD .....AC... ..F..S.... ....L..... RS......TN . [91]
#A/India/NIV-SARI-4571/2021_P2_PB1   .......... .......T.. .E...Q.... .....TQ... ....TTNQAD .....AC... ..F..S.... ....L..... RS......TN . [91]
```

```
#A/goose/Guangdong/1/1996          MEDFVRQCFN PMIVELAEKA MKEYGEDPKI ETNKFAAICT HLEVCFMYSD FHFIDERGES TIIESGDPNA LLKHRFEIIE GRDRTMAWTV VNSICNTTGV  [100]
#A/India/NIV-SARI-4571/2021_PA     .......... .......... .......... .......... .......... .......... I.V....... .......... .......... ......N...  [100]
#A/India/NIV-SARI-4571/2021_P1_PA  .......... .......... .......... .......... .......... .......... I.V....... .......... .......... ......N...  [100]
#A/India/NIV-SARI-4571/2021_P2_PA  .......... .......... .......... .......... .......... .......... I.V....... .......... .......... ......N...  [100]

#A/goose/Guangdong/1/1996          EKPKFLPDLY DYKENRFIEI GVTRREVHTY YLEKANKIKS EKTHIHIFSF TGEEMATKAD YTLDEESRAR IKTRLFTIRQ EMASRGLWDS FRQSERGEET  [200]
#A/India/NIV-SARI-4571/2021_PA     D......... ....S..... ........I. .......... .......... .......... .......... .......... .........P ..........  [200]
#A/India/NIV-SARI-4571/2021_P1_PA  D......... ....S..... ........I. .......... .......... .......... .......... .......... .........P ..........  [200]
#A/India/NIV-SARI-4571/2021_P2_PA  D......... ....S..... ........I. .......... .......... .......... .......... .......... .........P ..........  [200]

#A/goose/Guangdong/1/1996          VEERFEITGT MCRLADQSLP PNFSSLEKFR AYVDGFEPNG CIEGKLSQMS KEVNARIEPF LKTTPRPLRL PDGPPCSQRS KFLLMDALKL SIEDPSHEGE  [300]
#A/India/NIV-SARI-4571/2021_PA     I......... .R........ ......GN.. ......K... .......... .T.S...... .......... .......... .......... ..........  [300]
#A/India/NIV-SARI-4571/2021_P1_PA  I......... .R........ ......GN.. ......K... .......... .T.S...... .......... .......... .......... ..........  [300]
#A/India/NIV-SARI-4571/2021_P2_PA  I......... .R........ ......GN.. ......K... .......... .T.S...... .......... .......... .......... ..........  [300]

#A/goose/Guangdong/1/1996          GIPLYDAIKC MKTFFGWKEP NIVKPHEKGI NPNYLLAWKQ VLAELQDIEN EEKIPKTKNM RKTSQLKWAL GENMAPEKVD FEDCKDVSDL RQYDSDEPKP  [400]
#A/India/NIV-SARI-4571/2021_PA     .......... .......... ....S..... .......... .......... .......... K......... .......... .......... ........ES  [400]
#A/India/NIV-SARI-4571/2021_P1_PA  .......... .......... ....S..... .......... .......... .......... K......... .......... .......... ........ES  [400]
#A/India/NIV-SARI-4571/2021_P2_PA  .......... .......... ....S..... .......... .......... .......... K......... .......... .......... ........ES  [400]

#A/goose/Guangdong/1/1996          RSLASWIQSE FNKACELTDS SWIELDEIGE DVAPIEHIAS MRRNYFTAEV SHCRATEYIM KGVYINTALL NASCAAMDDF QLIPMISKCR TKEGRRKTNL  [500]
#A/India/NIV-SARI-4571/2021_PA     .......... .......... .......... .......... .......... .......... .......... .......... .......... ..........  [500]
#A/India/NIV-SARI-4571/2021_P1_PA  .......... .......... .......... .......... .......... .......... .......... .......... .......... ..........  [500]
#A/India/NIV-SARI-4571/2021_P2_PA  .......... .......... .......... .......... .......... .......... .......... .......... .......... ..........  [500]

#A/goose/Guangdong/1/1996          YGFLIKGRSH LRNDTDVVNF VSMEFSLTDP RLEPHRWEKY CVLRIGDMLL RTEIGQVSRP MFLYVRTNGT SKIKMKWGME MRRCPFQSLQ QIESMIEAES  [600]
#A/India/NIV-SARI-4571/2021_PA     ...I...... .......... .......... .....K.... ...E...... ..A....... .......... .......... ....LL.... ..........  [600]
#A/India/NIV-SARI-4571/2021_P1_PA  ...I...... .......... .......... .....K.... ...E...... ..A....... .......... .......... ....LL.... ..........  [600]
#A/India/NIV-SARI-4571/2021_P2_PA  ...I...... .......... .......... .....K.... ...E...... ..A....... .......... .......... ....LL.... ..........  [600]

#A/goose/Guangdong/1/1996          SVKEKDMTKE FFENKSETWP IGESPKGVEE GSIGKVCRTL LAKSVFNSLY ASPQLEGFSA ESRKLLLIVQ ALRDNLEPGT FDLGGLYEAI EECLINDPWV  [700]
#A/India/NIV-SARI-4571/2021_PA     ......V... .......... .......... .......... ..R....... .......... .......... .......... .......... ..........  [700]
#A/India/NIV-SARI-4571/2021_P1_PA  ......V... .......... .......... .......... ..R....... .......... .......... .......... .......... ..........  [700]
#A/India/NIV-SARI-4571/2021_P2_PA  ......V... .......... .......... .......... ..R....... .......... .......... .......... .......... ..........  [700]

#A/goose/Guangdong/1/1996          LLNASWFNSF LTHALR* [717]
#A/India/NIV-SARI-4571/2021_PA     .......... .....K. [717]
#A/India/NIV-SARI-4571/2021_P1_PA  .......... .....K. [717]
#A/India/NIV-SARI-4571/2021_P2_PA  .......... .....K. [717]
```

```
#A/goose/Guangdong/1/1996|0        DQICIGYHAN NSTEQVDTIM EKNVTVTHAQ DILEKTHNGK LCDLNGVKPL ILRDCSVAGW LLGNPMCDEF INVPEWSYIV EKASPANDLC YPGDFNDYEE  [100]
#A/India/NIV-SARI-4571/2021_HA     .H........ ...K...... .......... .......... .......... ..K....... .......... .......... ...N...G.. ...S......  [100]
#A/India/NIV-SARI-4571/2021_P1_HA  .H........ ...K...... .......... .......... .......... ..K....... .......... .......... ...N...G.. ...S......  [100]
#A/India/NIV-SARI-4571/2021_P2_HA  .H........ ...K...... .......... .......... .......... ..K....... .......... .......... ...N...G.. ...S......  [100]

#A/goose/Guangdong/1/1996|0        LKHLLSRTNH FEKIQIIPKS SWSNHDASSG VSSACPYHGR SSFFRNVVWL IKKNSAYPTI KRSYNNTNQE DLLVLWGIHH PNDAAEQTKL YQNPTTYISV  [200]
#A/India/NIV-SARI-4571/2021_HA     .......I.. ...VR....E ...E.EV.L. ..A....Q.N P......... ....NT.... .K........ ...I...... ..NE....N. .........I  [200]
#A/India/NIV-SARI-4571/2021_P1_HA  .......I.. ...VR....E ...E.EV.L. ..A....Q.N P......... ....NT.... .K........ ...I...... ..NE....N. .........I  [200]
#A/India/NIV-SARI-4571/2021_P2_HA  .......I.. ...VR....E ...E.EV.L. ..A....Q.N P......... ....NT.... .K........ ...I...... ..NE....N. .........I  [200]

#A/goose/Guangdong/1/1996|0        GTSTLNQRLV PEIATRPKVN GQSGRMEFFW TILKPNDAIN FESNGNFIAP EYAYKIVKKG DSAIMKSELE YGNCNTKCQT PMGAINSSMP FHNIHPLTIG  [300]
#A/India/NIV-SARI-4571/2021_HA     .......... .K....S.I. .....ID... ...N.....H .......... .......... ..T.....V. ......R... .I........ ..........  [300]
#A/India/NIV-SARI-4571/2021_P1_HA  .......... .K....S.I. .....ID... ...N.....H .......... .......... ..T.....V. ......R... .I........ ..........  [300]
#A/India/NIV-SARI-4571/2021_P2_HA  .......... .K....S.I. .....ID... ...N.....H .......... .......... ..T.....V. ......R... .I........ ..........  [300]

#A/goose/Guangdong/1/1996|0        ECPKYVKSNR LVLATGLRNT PQRERRRKKR GLFGAIAGFI EGGWQGMVDG WYGYHHSNEQ GSGYAADKES TQKAIDGVTN KVNSIIDKMN TQFEAVGREF  [400]
#A/India/NIV-SARI-4571/2021_HA     .........K .........S ........-. .......... .......... .......... .......... .......... .......... ..........  [400]
#A/India/NIV-SARI-4571/2021_P1_HA  .........K .........S ........-. .......... .......... .......... .......... .......... .......... ..........  [400]
#A/India/NIV-SARI-4571/2021_P2_HA  .........K .........S ........-. .......... .......... .......... .......... .......... .......... ..........  [400]

#A/goose/Guangdong/1/1996|0        NNLERRIENL NKQMEDGFLD VWTYNAELLV LMENERTLDF HDSNVKNLYD KVRLQLRDNA KELGNGCFEF YHKCDNECME SVKNGTYDYP QYSEEARLNR  [500]
#A/India/NIV-SARI-4571/2021_HA     .R........ ..K....... .......... .......... .....R.... ......K... ..M....... .......... ..R....... .....S..K.  [500]
#A/India/NIV-SARI-4571/2021_P1_HA  .R........ ..K....... .......... .......... .....R.... ......K... ..M....... .......... ..R....... .....S..K.  [500]
#A/India/NIV-SARI-4571/2021_P2_HA  .R........ ..K....... .......... .......... .....R.... ......K... ..M....... .......... ..R....... .....S..K.  [500]

#A/goose/Guangdong/1/1996|0        EEISGVKLES MGTYQILSIY STVASSLALA IMVAGLSLWM CSNGSLQCRI CI* [553]
#A/India/NIV-SARI-4571/2021_HA     .........P I.I....... .......V.. ..M....... .......... ... [553]
#A/India/NIV-SARI-4571/2021_P1_HA  .........P I.I....... .......V.. ..M....... .......... ... [553]
#A/India/NIV-SARI-4571/2021_P2_HA  .........P I.I....... .......V.. ..M....... .......... ... [553]
```

```
#A/goose/Guangdong/1/1996          MASQGTKRSY EQMETGGERQ NATEIRASVG RMVGGIGRFY IQMCTELKLS DYEGRLIQNS ITIERMVLSA FDERRNKYLE EHPSAGKDPK KTGGPIYRRR  [100]
#A/India/NIV-SARI-4571/2021_NP     .......... .......... .......... .......... V......Q.. .......... .......... ......R... .......... ..........  [100]
#A/India/NIV-SARI-4571/2021_P1_NP  .......... .......... .......... .......... V......Q.. .......... .......... ......R... .......... ..........  [100]
#A/India/NIV-SARI-4571/2021_P2_NP  .......... .......... .......... .......... V......Q.. .......... .......... ......R... .......... ..........  [100]

#A/goose/Guangdong/1/1996          DGKWVRELIL YDKEEIRRIW RQANNGEDAT AGLTHMMIWH SNLNDATYQR TRALVRTGMD PRMCSLMQGS TLPRRSGAAG AAVKGVGTMV MELIRMIKRG  [200]
#A/India/NIV-SARI-4571/2021_NP     ....M..... .......... .......... .....L.... .....T.... .......... .......... .......... .......... ..........  [200]
#A/India/NIV-SARI-4571/2021_P1_NP  ....M..... .......... .......... .....L.... .....T.... .......... .......... .......... .......... ..........  [200]
#A/India/NIV-SARI-4571/2021_P2_NP  ....M..... .......... .......... .....L.... .....T.... .......... .......... .......... .......... ..........  [200]

#A/goose/Guangdong/1/1996          INDRNFWRGE NGRRTRIAYE RMCNILKGKF QTAAQRAMMD QVRESRNPGN AEIEDLIFLA RSALILRGSV AHKSCLPACV YGLAVASGYD FEREGYSLVG  [300]
#A/India/NIV-SARI-4571/2021_NP     .......... .......... .......... .......... .......... .......... .......... .......... .......... ..........  [300]
#A/India/NIV-SARI-4571/2021_P1_NP  .......... .......... .......... .......... .......... .......... .......... .......... .......... ..........  [300]
#A/India/NIV-SARI-4571/2021_P2_NP  .......... .......... .......... .......... .......... .......... .......... .......... .......... ..........  [300]

#A/goose/Guangdong/1/1996          IDPFRLLQNS QVFSLIRPNE NPAHKSQLVW MACHSAAFED LRVSSFIRGT RVAPRGQLST RGVQIASNEN METMDSSTLE LRSRYWAIRT RSGGNTNQQR  [400]
#A/India/NIV-SARI-4571/2021_NP     .......... .......... .......... .......... .......... ..V....... .......... ..A....... .......... ..........  [400]
#A/India/NIV-SARI-4571/2021_P1_NP  .......... .......... .......... .......... .......... ..V....... .......... ..A....... .......... ..........  [400]
#A/India/NIV-SARI-4571/2021_P2_NP  .......... .......... .......... .......... .......... ..V....... .......... ..A....... .......... ..........  [400]

#A/goose/Guangdong/1/1996          ASAGQISVQP TFSVQRNLPF ERATIMAAFT GNTEGRTSDM RTEIIRMMES SRPEDVSFQG RGVFELSDEK ATNPIVPSFD MSNEGSYFFG DNAEEYDN* [499]
#A/India/NIV-SARI-4571/2021_NP     .......... A......... .......... .......... .......... A......... .......... .......... .......... ......... [499]
#A/India/NIV-SARI-4571/2021_P1_NP  .......... A......... .......... .......... .......... A......... .......... .......... .......... ......... [499]
#A/India/NIV-SARI-4571/2021_P2_NP  .......... A......... .......... .......... .......... A......... .......... .......... .......... ......... [499]
```

```
#A/goose/Guangdong/1/1996|0        MNPNQKIITI GSICMVVGII SLMLQIGNII SIWVSHSIQT GNQHQAEPCN QSIITYENNT WVNQTYVNIS NTNFLTEKAV ASVTLAGNSS LCPISGWAVH  [100]
#A/India/NIV-SARI-4571/2021_NA     .......M.. .LV...L..V ..I.....T. LM..R..F.I EA.....--. .--------- ---------- .A...N.N.. ...A.V.... ....R.....  [100]
#A/India/NIV-SARI-4571/2021_P1_NA  .......M.. .LV...L..V ..I.....T. LM..R..F.I EA.....--. .--------- ---------- .A...N.N.. ...A.V.... ....R.....  [100]
#A/India/NIV-SARI-4571/2021_P2_NA  .......M.. .LV...L..V ..I.....T. LM..R..F.I EA........ .--------- ---------- .A...N.N.. ...A.V.... ....R.....  [100]

#A/goose/Guangdong/1/1996|0        SKDNGIRIGS KGDVFVIREP FISCSHLECR TFFLTQGALL NDKHSNGTVK DRSPHRTLMS CPVGEAPSPY NSRFESVAWS ASACHDGTSW LTIGISGPDN  [200]
#A/India/NIV-SARI-4571/2021_NA     ....S..... .......... .......... .......... .......... .......... ..L....... .......... .......... .........D  [200]
#A/India/NIV-SARI-4571/2021_P1_NA  ....S..... .......... .......... .......... .......... .......... ..L....... .......... .......... .........D  [200]
#A/India/NIV-SARI-4571/2021_P2_NA  ....S..... .......... .......... .......... .......... .......... ..L....... .......... .......... .........D  [200]

#A/goose/Guangdong/1/1996|0        GAVAVLKYNG IITDTIKSWR NNILRTQESE CACVNGSCFT VMTDGPSNGQ ASYKIFKMEK GKVVKSVELN APNYHYEECS CYPDAGEITC VCRDNWHGSN  [300]
#A/India/NIV-SARI-4571/2021_NA     .......... .....V.... .......... .......... .......... .......VK. .......... .......... ........M. ....S.....  [300]
#A/India/NIV-SARI-4571/2021_P1_NA  .......... .....V.... .......... .......... .......... .......VK. .......... .......... ........M. ....S.....  [300]
#A/India/NIV-SARI-4571/2021_P2_NA  .......... .....V.... .......... .......... .......... .......VK. .......... .......... ........M. ....S.....  [300]

#A/goose/Guangdong/1/1996|0        RPWVSFNQNL EYQIGYICSG VFGDNPRPND GTGSCGPVSP NGAYGVKGFS FKYGNGVWIG RTKSTNSRSG FEMIWDPNGW TGTDSSFSVK QDIVAITDWS  [400]
#A/India/NIV-SARI-4571/2021_NA     .......... .......... .......... .........S .......... .......... .......... ...V...... .D...N..I. ..........  [400]
#A/India/NIV-SARI-4571/2021_P1_NA  .......... .......... .......... .........S .......... .......... .......... ...V...... .D...N..I. ..........  [400]
#A/India/NIV-SARI-4571/2021_P2_NA  .......... .......... .......... .........S .......... .......... .......... ...V...... .D...N..I. ..........  [400]

#A/goose/Guangdong/1/1996|0        GYSGSFVQHP ELTGLDCIRP CFWVELIRGR PKESTIWTSG SSISFCGVNS DTVGWSWPDD AELPFTIDK*  [470]
#A/India/NIV-SARI-4571/2021_NA     .......... .......... ...I...... .......... .......... E..S.....G ..........  [470]
#A/India/NIV-SARI-4571/2021_P1_NA  .......... .......... ...I...... .......... .......... E..S.....G ..........  [470]
#A/India/NIV-SARI-4571/2021_P2_NA  .......... .......... ...I...... .......... .......... E..S.....G ..........  [470]
```

```
#A/goose/Guangdong/1/1996          MSLLTEVETY VLSIVPSGPL KAEIAQRLED VFAGKNTDLE ALMEWLKTRP ILSPLTKGIL GFVFTLTVPS ERGLQRRRFV QNALNGNGDP NNMDRAVKLY  [100]
#A/India/NIV-SARI-4571/2021_MP     .......... ....I..... ......K... .......... .......... .......... .......... .......... ..S....... ..........  [100]
#A/India/NIV-SARI-4571/2021_P1_MP  .......... ....I..... ......K... .......... .......... .......... .......... .......... ..S....... ..........  [100]
#A/India/NIV-SARI-4571/2021_P2_MP  .......... ....I..... ......K... .......... .......... .......... .......... .......... ..S....... ..........  [100]

#A/goose/Guangdong/1/1996          KKLKREITFH GAKEVALSYS TGALASCMGL IYNRMGTVTT EVAFGLVCAT CEQIADSQHR SHRQMATTTN PLIRHENRMV LASTTAKAME QMAGSSEQAA  [200]
#A/India/NIV-SARI-4571/2021_MP     .......... .......... .......... .......... .......... .......... .......I.. .......... .......... ..........  [200]
#A/India/NIV-SARI-4571/2021_P1_MP  .......... .......... .......... .......... .......... .......... .......I.. .......... .......... ..........  [200]
#A/India/NIV-SARI-4571/2021_P2_MP  .......... .......... .......... .......... .......... .......... .......I.. .......... .......... ..........  [200]

#A/goose/Guangdong/1/1996          EAMEVASQAR QMVQAMRTIG THPSSSAGLK DNLLENLQAY QKRMGVQMQR FK* [253]
#A/India/NIV-SARI-4571/2021_MP     ....I.N... .......... ...N.....R .......... .......... ... [253]
#A/India/NIV-SARI-4571/2021_P1_MP  ....I.N... .......... ...N.....R .......... .......... ... [253]
#A/India/NIV-SARI-4571/2021_P2_MP  ....I.N... .......... ...N.....R .......... .......... ... [253]
```

```
#A/goose/Guangdong/1/1996          PTKNEWECKC SDSSDPLVVA ASIIGILHLI LWILDRLFFK CIYRRLKYGL KRGPSTEGVP ESMREEYRQE QQSAVDVDDG HFVNIELE*
#A/India/NIV-SARI-4571/2021_MP     H.R.....R. .........T .......... .......... .......... ......K... .......... .......... .........
#A/India/NIV-SARI-4571/2021_P1_MP  H.R.....R. .........T .......... .......... .......... ......K... .......... .......... .........
#A/India/NIV-SARI-4571/2021_P2_MP  H.R.....R. .........T .......... .......... .......... ......K... .......... .......... .........
```

```
#A/goose/Guangdong/1/1996           MDSNTITSFQ VDCYLWHIRK LLSMRDMCDA PFDDRLRRDQ KALKGRGSTL GLDLRVATME GKKIVEDILK SETNENLKIA IASSPAPRYI TDMSIEEMSR [100]
#A/India/NIV-SARI-4571/2021_NS      .......... .......... .......... .......... .......... .......... .......... ...D...... .......... .......I..
#A/India/NIV-SARI-4571/2021_P1_NS   .......... .......... .......... .......... .......... .......... .......... ...D...... .......... .......I..
#A/India/NIV-SARI-4571/2021_P2_NS   .......... .......... .......... .......... .......... .......... .......... ...D...... .......... .......I..

#A/goose/Guangdong/1/1996           EWYMLMPRQK ITGGLMVKMD QAIMDKRIIL KANFSVLFDQ LETLVSLRAF TESGAIVAEI FPIPSVPGHF TEDVKNAIGI LIGGLEWNDN SIRASENIQR [200]
#A/India/NIV-SARI-4571/2021_NS      G.H.M..K.. .......... .......... .........R .......... ..D....... S....M...S .......... .......... ..........
#A/India/NIV-SARI-4571/2021_P1_NS   G.H.M..K.. .......... .......... .........R .......... ..D....... S....M...S .......... .......... ..........
#A/India/NIV-SARI-4571/2021_P2_NS   G.H.M..K.. .......... .......... .........R .......... ..D....... S....M...S .......... .......... ..........

#A/goose/Guangdong/1/1996           FAWGIHDENG GPSLPPKQKR YMAKRVESEV *
#A/India/NIV-SARI-4571/2021_NS      .....R.... ..P.L.E... ...R...... .
#A/India/NIV-SARI-4571/2021_P1_NS   .....R.... ..P.L.E... ...R...... .
#A/India/NIV-SARI-4571/2021_P2_NS   .....R.... ..P.L.E... ...R...... .


#A/goose/Guangdong/1/1996           QDILQRMSKM QLESSSVDLN GMITQFERLK IYRDSLGESM MRMGDLHSLQ NRNATWRNEL SQKFEEIRWL IAECRNILTK TENSFEQITF LQALQLLLEV
#A/India/NIV-SARI-4571/2021_NS      .......... .......... .......... .........V ........F. S......E.. .......... .......... .......... ..........
#A/India/NIV-SARI-4571/2021_P1_NS   .......... .......... .......... .........V ........F. S......E.. .......... .......... .......... ..........
#A/India/NIV-SARI-4571/2021_P2_NS   .......... .......... .......... .........V ........F. S......E.. .......... .......... .......... ..........

#A/goose/Guangdong/1/1996           ESEIRTFSFQ LI*
#A/India/NIV-SARI-4571/2021_NS      .......... ...
#A/India/NIV-SARI-4571/2021_P1_NS   .......... ...
#A/India/NIV-SARI-4571/2021_P2_NS   .......... ...
```

Appendix Figure 2. Amino acid alignment of 11 genes relative to A/goose/Guangdong/1/1996as prototype virus and the sequence obtained from the clinical sample and the passaged 1 and 2 virus of newly identified H5N1virus.
